# Comparing the Voets equation and the Adrogue-Madias equation for predicting the plasma sodium response to intravenous fluid therapy in SIADH patients

**DOI:** 10.1371/journal.pone.0245499

**Published:** 2021-01-15

**Authors:** Philip J. G. M. Voets, Nils P. J. Vogtländer, Karin A. H. Kaasjager

**Affiliations:** 1 Department of Nephrology, University Medical Centre Utrecht, Utrecht, The Netherlands; 2 Department of Nephrology, Gelre Hospital, Apeldoorn, The Netherlands; International University of Health and Welfare, School of Medicine, JAPAN

## Abstract

**Background:**

The syndrome of inappropriate antidiuretic hormone secretion (SIADH) is one of the most common causes of hypotonic hyponatremia. In our previous work, we have derived a novel model (Voets equation) that can be used by clinicians to predict the effect of crystalloid intravenous fluid therapy on the plasma sodium concentration in SIADH.

**Methods:**

In this retrospective chart review, the predictive accuracy of the Voets equation and the Adrogue-Madias equation for the plasma sodium response to crystalloid infusate was compared for fifteen plasma sodium response measurements (*n* = 15) in twelve SIADH patients. The medical records of these patients were accessed anonymously and none of the authors were their treating physicians. The Pearson correlation coefficient *r* and corresponding *p*-value were calculated for the predictions by the Voets model compared to the measured plasma sodium response and for the predictions by the Adrogue-Madias model compared to the measured plasma sodium response.

**Results and conclusion:**

The presented results show that the Voets model (*r* = 0.94, *p* < 0.001) predicted the aforementioned plasma sodium response significantly more accurately than the Adrogue-Madias model (*r* = 0.49, *p* = 0.07) in SIADH patients and could therefore be a clinically useful addition to the existing prediction models.

## 1. Introduction

The syndrome of inappropriate antidiuretic hormone secretion (SIADH) is one of the most common causes of hypotonic hyponatremia, which is defined as a plasma sodium concentration below 135 mmol/L in the context of plasma hypotonicity [1–4]. This condition is characterized by the feedback-independent–and often tonic–release of antidiuretic hormone (ADH) or arginine vasopressin, an oligopeptide hormone that stimulates the translocation of aquaporin 2 water channels in the collecting ducts and promotes pure water retention by the kidneys [1,2,4]. ADH can be secreted in this feedback-independent fashion by the posterior pituitary gland, often provoked by certain drugs or pain, or by malignant cells in the case of paraneoplastic SIADH, especially in patients with small-cell lung cancer [1,2,4]. Furthermore, SIADH can be mimicked by the exogenous administration of vasopressin analogues, such as desmopressin, in the treatment of–among other afflictions–enuresis nocturna or certain types of Von Willebrand disease [1,4]. Because the ADH release in SIADH patients is not governed by physiological osmotic stimuli, their renal ability to excrete water is greatly diminished–which is reflected by concentrated urine with a relatively elevated and fixed urine osmolality–whereas their renal ability to excrete sodium remains fairly unaffected [1,2,4]. As a result, it has classically been accepted by clinicians that administering normal saline infusate to a SIADH patient will exacerbate hypotonic hyponatremia and should thus be considered an inappropriate treatment strategy for these patients [1–3]. Some authors have even suggested that the exacerbation of hypotonic hyponatremia in response to administered normal saline should be considered a confirmation of SIADH [3].

In our previous work, we have expounded on this clinical dogma and we have presented a theoretical foundation for the opposing observations by–among others–Shimizu *et al*., Hoorn *et al*. and Zietse *et al*. that normal saline can in fact be effective in treating SIADH-induced hypotonic hyponatremia, as long as the urine is not concentrated beyond approximately 530 mOsmol/L [4–8]. Based on the electrolyte-free water balance of intravenous fluid input versus urine output, we have previously derived a novel model–hereafter referred to as the Voets equation for clarity–that can be used to predict the effect of various volumes of crystalloid infusate with various tonicities on the plasma sodium concentration in SIADH, taking into account patient characteristics, such as the total body water, the urine osmolality, and the initial plasma sodium concentration [4]. Using a retrospective chart review, we have experimentally validated our previously presented mathematical model in SIADH patients by comparing the plasma sodium response to crystalloid intravenous fluid therapy of varying volumes and tonicities as predicted by our model to the measured plasma sodium change. Furthermore, we have compared these predictions of our model to the plasma sodium change predictions by the widely used Adrogue-Madias equation [2,9].

Below, we present and discuss the results of this retrospective validation study.

## 2. Methods and results

Fifteen measurements of the plasma sodium response to saline infusate (*n* = 15) in twelve different SIADH patients from our clinic were documented. Their plasma sodium concentration before and after administering the saline infusate ([*Na*^+^]_*p*,1_ and [*Na*^+^]_*p*,2_, respectively) was recorded from their patient files. The aforementioned patients were included retrospectively, as we strongly felt that it would be unethical to deliberately administer a treatment which might potentially exacerbate a pre-existing hyponatremia. The medical records of these patients were accessed anonymously and none of the authors were their treating physicians.

The measured difference in plasma sodium concentration (Δ[*Na*^+^]_*p*_) was compared to the change in the plasma sodium concentration predicted by the Voets equation and the Adrogue-Madias equation and the Pearson correlation coefficient *r* was calculated for both prediction models. Our calculations showed that in order to detect an estimated and rather conservative correlation of *r* = 0.70, using a two-sided test, a 5% significance level (*α* = 0.05), and a statistical power of 80% (*β* = 0.20), the required sample size would be approximately thirteen (*n* = 13). Patient characteristics are summarized in Table 1. If multiple volumes of saline infusate had been administered to the same SIADH patient (which was the case in three patients), the results are presented as separate measurements and thus as separate patients in Table 2. The average time between the first and second measurement of the plasma sodium concentration was approximately six hours. If plasma sodium measurements are performed too soon after each other, the kidneys will not have had sufficient time able to process the introduced saline. The diagnosis of SIADH was made based largely on the original Bartter-Schwartz criteria (i.e., hypotonic hyponatremia, urine that is not maximally diluted (higher than 100 mOsmol/L), clinical euvolemia, no hypothyroidism or adrenal insufficiency, and no diuretic use reported by the patients) [1]. In order to further establish SIADH as the most likely cause of hypotonic hyponatremia, only patients with a urine osmolality higher than their plasma osmolality were included, as we felt that this was a strong argument in favor of significant inappropriate ADH release. If possible, a causative underlying condition for the SIADH was identified. The administered saline volumes ranged from 0.10 liters to 1.5 liters and tonicities ranged from 308 mmol/L (0.9%-NaCl) to 856 mmol/L (2.5%-NaCl). Below, the Voets equation is presented as Eq (1):

$$\Delta {\left[{Na}^{+}\right]}_{p}=\frac{{\left[{Na}^{+}\right]}_{p}V_{i}}{TBW}\left(1.7\frac{O_{i}}{O_{u}}-1\right)$$ (1)

**Table 1:** Characteristics of the included SIADH patients and the administered intravenous fluids.

Measurement	TBW (weight)	Infusate (*V*_*i*_, type)	[*Na*^+^]_*p*,1_	[*Na*^+^]_*p*,2_	*O*_*u*_
1 (M, 79 y.o.)	34 L (57 kg)	1.5L 0.9%-NaCl	133 mmol/L	131 mmol/L	766 mOsmol/L
2 (M, 78 y.o.)	37 L (62 kg)	1.0 L 0.9%-NaCl	132 mmol/L	131 mmol/L	702 mOsmol/L
3 (M, 74 y.o.)	30 L (60 kg)	1.0 L 0.9%-NaCl	130 mmol/L	129 mmol/L	614 mOsmol/L
4 (M, 74 y.o.)	30 L (60 kg)	0.5 L 0.9%-NaCl	129 mmol/L	128 mmol/L	689 mOsmol/L
5 (M, 59 y.o.)	40 L (77 kg)	1.0 L 0.9%-NaCl	129 mmol/L	128 mmol/L	890 mOsmol/L
6 (F, 66 y.o.)	33 L (66 kg)	1.0 L 0.9%-NaCl	131 mmol/L	130 mmol/L	569 mOsmol/L
7 (F, 78 y.o.)	25 L (50 kg)	1.0 L 0.9%-NaCl	128 mmol/L	127 mmol/L	660 mOsmol/L
8 (F, 82 y.o.)	27 L (54 kg)	1.5 L 0.9%-NaCl	128 mmol/L	128 mmol/L	496 mOsmol/L
9 (F, 78 y.o.)	25 L (50 kg)	0.10 L 2.5%-NaCl	127 mmol/L	128 mmol/L	677 mOsmol/L
10 (M, 59 y.o.)	60 L (100 kg)	0.15 L 2.5%-NaCl	106 mmol/L	107 mmol/L	336 mOsmol/L
11 (F, 87 y.o.)	43 L (86 kg)	1.5 L 0.9%-NaCl	131 mmol/L	132 mmol/L	386 mOsmol/L
12 (M, 69 y.o.)	47 L (79 kg)	0.15 L 2.5%-NaCl	122 mmol/L	123 mmol/L	645 mOsmol/L
13 (M, 69 y.o.)	47 L (79 kg)	0.10 L 2.5%-NaCl	123 mmol/L	124 mmol/L	559 mOsmol/L
14 (F, 84 y.o.)	35 L (70 kg)	0.10 L 2.5%-NaCl	121 mmol/L	122 mmol/L	354 mOsmol/L
15 (F, 85 y.o.)	29 L (58 kg)	0.15 L 2.5%-NaCl	122 mmol/L	124 mmol/L	345 mOsmol/L

**Table 2:** Comparison of the Voets (V) equation and the modified Adrogue-Madias (A-M) equation for prediction of the change in plasma sodium concentration in response to intravenous fluid therapy in SIADH patients.

*n*	Measured Δ[*Na*^+^]_*p*_	Predicted Δ[*Na*^+^]_*p*_ according to V*	Predicted Δ[*Na*^+^]_*p*_ according to A-M**	SIADH	Causative condition
1	-2 mmol/L	-1.9 mmol/L	+0.9 mmol/L	Yes	Citalopram use
2	-1 mmol/L	-1.2 mmol/L	+0.6 mmol/L	Yes	Pain due to leg ischemia
3	-1 mmol/L	-0.8 mmol/L	+0.8 mmol/L	Yes	Pneumonia
4	-1 mmol/L	-0.5 mmol/L	+0.4 mmol/L	Yes	Pneumonia
5	-1 mmol/L	-1.7 mmol/L	+0.6 mmol/L	Yes	Quetiapine use
6	-1 mmol/L	-0.4 mmol/L	+0.7 mmol/L	Yes	Non-small cell lung carcinoma
7	-1 mmol/L	-1.3 mmol/L	+1.0 mmol/L	Yes	Pain due to hip fracture
8	0 mmol/L	+0.4 mmol/L	+1.4 mmol/L	Yes	Unknown
9	+1 mmol/L	+0.6 mmol/L	+1.2 mmol/L	Yes	Pain due to hip fracture
10	+1 mmol/L	+0.9 mmol/L	+0.8 mmol/L	Yes	Pneumonia
11	+1 mmol/L	+1.6 mmol/L	+0.8 mmol/L	Yes	Unknown
12	+1 mmol/L	+0.5 mmol/L	+1.0 mmol/L	Yes	Psychosis
13	+1 mmol/L	+0.4 mmol/L	+0.6 mmol/L	Yes	Psychosis
14	+1 mmol/L	+1.1 mmol/L	+0.9 mmol/L	Yes	Pneumonia
15	+2 mmol/L	+2.0 mmol/L	+1.6 mmol/L	Yes	Viral respiratory tract infection

In order to correct for varying infusate volumes, the Adrogue-Madias equation was algebraically modified, as the original equation can only be used for 1.0 liter of administered infusate (see Appendix) [2]. Hereafter, Eq (2) will be referred to as the (modified) Adrogue-Madias equation:

$$\Delta {\left[{Na}^{+}\right]}_{p}=\frac{V_{i}({\left[{Na}^{+}\right]}_{i}-{\left[{Na}^{+}\right]}_{p})}{TBW+V_{i}}$$ (2)

**Fig 1:**
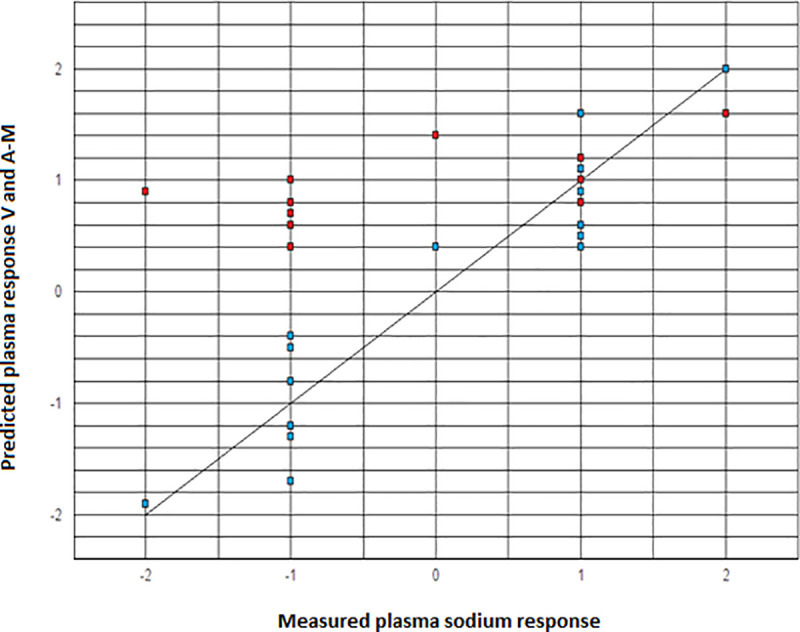


The Pearson correlation coefficient *r* (*r* = 0.94 for the predictions by the Voets model compared to the measured plasma sodium response versus *r* = 0.49 for the predictions by the Adrogue-Madias model compared to the measured plasma sodium response) and the corresponding *p*-values (*p* < 0.001 for Voets model versus *p* = 0.07 for Adrogue-Madias model) were calculated and the correlation scatter plots were presented (see Fig 1) [10].

### Informed consent/Ethical approval

Not applicable for retrospective chart review; the medical records of these patients were accessed anonymously and none of the authors were their treating physicians.

## 3. Discussion

In the previous section we have compared the predictive accuracy of the Voets equation and the Adrogue-Madias equation for the plasma sodium response to crystalloid infusate for fifteen plasma sodium response measurements in SIADH patients from our clinic [2,4,9]. The presented results show that the Voets model (*r* = 0.94, *p* < 0.001) predicted this plasma sodium response significantly more accurately than the Adrogue-Madias model (*r* = 0.49, *p* = 0.07) in SIADH patients. When comparing our model to the one presented by Adrogue-Madias, a rather intuitive Bayesian principle seems to apply, in which predictions tend to become more accurate as more information is included in the model. This was especially true when a decrease in the plasma sodium concentration occurred in response to intravenous fluid therapy, whereas both equations were relatively comparable in predictive accuracy for increases in the plasma sodium concentrations. This discrepancy can be explained by the fact that the Voets model takes both infusate input and urine output into account and therefore provides a more accurate overall prediction of the plasma sodium response, as opposed to the Adrogue-Madias equation which solely considers redistribution of the introduced infusate [2,4,9]. The more concentrated the urine of these SIADH patients (which generally reflects a stronger release of ADH) and thus the lower their electrolyte-free water excretion, the stronger the effect of output–rather than input–on their overall electrolyte-free water balance and therefore on their net plasma sodium response to intravenous fluid therapy [4,5,8]. In our opinion, including the urine output parameter in our equation considerably increases its predictive accuracy, but does not significantly add to its complexity and therefore does not limit its clinical applicability [4]. Although we feel that the assumption of relatively fixed urine osmolality in SIADH (classically considered a hallmark of this condition) is reasonable for the purpose of deriving our prediction model, this is not always true, as more than one ADH release pattern has been described in SIADH [1]. It also stands to reason that the stimulus or agent which provokes aberrant ADH release can be removed, in which case SIADH ceases to exist and our model can no longer be reliably used.

The limitations of the Adrogue-Madias equation, which we have also discussed in this article, and comparable prediction models have previously been noted by several authors [11–13]. At the same time, these authors also express the strong desire among physicians for a clinical equation that can accurately predict the plasma sodium response to intravenous fluid therapy [11–13]. The daunting mathematical complexity of some existing models is another factor that can limit their clinical utility, which is a major concern with prediction equations such as the one proposed by Nguyen and Kurtz [13,14]. Since the Voets equation is primarily intended for a quick ‘bed-side evaluation’ of the effect of intravenous fluid therapy on the plasma sodium concentration in SIADH patients, mathematical transparency should be considered a *condicio sine qua non*. Furthermore, our model can be used to predict the plasma sodium response to varying volumes and tonicities of crystalloid infusate, rather than the standard 1.0 liter of crystalloid infusate in the original Adrogue-Madias equation [2,9]. For the purpose of our model, other sources of fluid input and/or output, such as insensible water losses or diarrhea, were not taken into account [4]. If such fluid gains or losses are significant, this should be considered a potential source of error.

The most important limitation of our equation is that it can only be reliably applied to SIADH patients [4]. Although we strongly suspect that our model could also be applied to patients with other types of feedback-independent ADH release (such as diabetes insipidus or reset osmostat syndrome) and to patients receiving continuous administration of vasopressin analogues (most notably, Intensive Care patients with circulatory shock or patients with severe hyponatremia who are treated with a so-called “desmopressin (DDAVP) clamp strategy” to prevent rapid auto-correction of the plasma sodium concentration), these have not been included in our analysis [4,15]. The reason for this limitation is that our model assumes that the urine osmolality in SIADH patients, which we have used as a measure for the theoretical maximum urine tonicity in our previous work, does not change to a relevant extent between the two measurements of the plasma sodium concentration [4,5,16]. This is a reasonable assumption in the case of relatively tonic, feedback-independent ADH release, but not for many other causes of dysnatremia. When applying our model to guide intravenous fluid therapy in hypotonic hyponatremia, the clinician should verify that SIADH is the most likely causative disorder. Furthermore, our equation has not been validated in patients receiving non-crystalloid intravenous fluids (e.g., intravenous sugar solutions) [4]. However, since it seems highly unlikely that a patient suffering from hypotonic hyponatremia would receive non-crystalloid infusate, this does not seem to be a clinically important limitation. Due to the retrospective nature of this validation study, our model was tested in a relatively small group of SIADH patients. Further (prospective) validation of our prediction model in a larger group of SIADH patients remains desirable. Ideally, such a validation study should also aim to expand the application of our model to other disorders that are characterized by feedback-independent ADH release, but this falls beyond the scope of this article.

In conclusion, we believe that the Voets equation is a mathematically transparent clinical tool to accurately guide intravenous fluid therapy in patients suffering from SIADH, which remains one of the most common causes of hypotonic hyponatremia. This being said, no mathematical model is incontrovertible. Frequent measurements of the plasma sodium concentration and astute clinical reasoning by the attending physician remain imperative.

## 4. Appendix

In order to correct for varying infusate volumes, the Adrogue-Madias equation was algebraically modified, as the original equation (Eq 3.1) can only be used for 1.0 liter of infusate [2,9]:

$$\Delta {\left[{Na}^{+}\right]}_{p}=\frac{{\left[{Na}^{+}\right]}_{i}-{\left[{Na}^{+}\right]}_{p}}{TBW+1}$$ (3.1)

The plasma sodium change (Δ[*Na*^+^]_*p*_) to infusate (with volume *V*_*i*_ and sodium concentration [*Na*^+^]_*i*_) can be calculated by subtracting the original plasma sodium concentration ([*Na*^+^]_*p*,1_) from the plasma sodium concentration after the infusate has been administered ([*Na*^+^]_*p*,2_):

$$\Delta {\left[{Na}^{+}\right]}_{p}={\left[{Na}^{+}\right]}_{p,2}-{\left[{Na}^{+}\right]}_{p,1}=\frac{{{Na}_{p}}^{+}+{{Na}_{i}}^{+}}{TBW+V_{i}}-\frac{{{Na}_{p}}^{+}}{TBW}$$ (3.2)

In order to improve mathematical clarity and because this retrospective validation study looks at different types saline (which is free of potassium), potassium is disregarded in Eq (3.2). This equation can be algebraically rewritten to:

$$\begin{array}{c} \frac{TBW({{Na}_{p}}^{+}+{{Na}_{i}}^{+})}{TBW(TBW+V_{i})}-\frac{{{Na}_{p}}^{+}(TBW+V_{i})}{TBW(TBW+V_{i})}=\\ \frac{{TBW{Na}_{p}}^{+}+{TBW{Na}_{i}}^{+}-{TBW{Na}_{p}}^{+}-V_{i}{{Na}_{p}}^{+}}{TBW(TBW+V_{i})}=\\ \frac{{TBW{Na}_{i}}^{+}-V_{i}{{Na}_{p}}^{+}}{TBW(TBW+V_{i})}=\frac{{{Na}_{i}}^{+}-V_{i}({{Na}_{p}}^{+}/TBW)}{TBW+V_{i}} \end{array}$$ (3.3)

This produces the modified Adrogue-Madias equation, which was used in our article:

$$\frac{V_{i}{\left[{Na}^{+}\right]}_{i}-V_{i}({{Na}_{p}}^{+}/TBW)}{TBW+V_{i}}=\frac{V_{i}{\left[{Na}^{+}\right]}_{i}-V_{i}{\left[{Na}^{+}\right]}_{p}}{TBW+V_{i}}=\frac{V_{i}({\left[{Na}^{+}\right]}_{i}-{\left[{Na}^{+}\right]}_{p})}{TBW+V_{i}}$$ (3.4)
