# Correction: Comparing the Voets equation and the Adrogue-Madias equation for predicting the plasma sodium response to intravenous fluid therapy in SIADH patients

**DOI:** 10.1371/journal.pone.0248445

**Published:** 2021-03-04

**Authors:** Philip J. G. M. Voets, Nils P. J. Vogtländer, Karin A. H. Kaasjager

The caption for Fig 1 is missing. Please see the complete, correct Fig 1 caption here.

**Fig 1:**
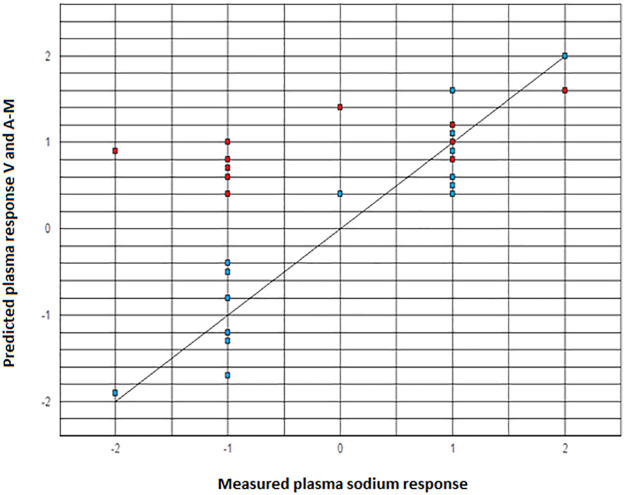
Scatter plot showing a comparison of the Voets (V) equation (blue dots) and the modified Adrogue-Madias (A-M) equation (red dots) for prediction of the change in plasma sodium concentration in response to intravenous fluid therapy in the included SIADH patients (scatter plot was created using www.onlinecharttool.com)^[10]^.

## References

[1] Voets PJGM, Vogtländer NPJ, Kaasjager KAH (2021) Comparing the Voets equation and the Adrogue-Madias equation for predicting the plasma sodium response to intravenous fluid therapy in SIADH patients. PLoS ONE 16(1): e0245499. 10.1371/journal.pone.0245499 33449937 PMC7810276

